# Development of Genetically Flexible Mouse Models of Sarcoma Using RCAS-TVA Mediated Gene Delivery

**DOI:** 10.1371/journal.pone.0094817

**Published:** 2014-04-14

**Authors:** Leah Kabaroff, Amar Gupta, Serena Menezes, Yael Babichev, Rita C. Kandel, Carol J. Swallow, Brendan C. Dickson, Rebecca A. Gladdy

**Affiliations:** 1 Lunenfeld-Tanenbaum Research Institute, Mount Sinai Hospital, Toronto, Ontario, Canada; 2 Institute of Medical Science, University of Toronto, Toronto, Ontario, Canada; 3 Department of Laboratory Medicine and Pathobiology, University of Toronto, Toronto, Ontario, Canada; 4 Department of Pathology and Laboratory Medicine, Mount Sinai Hospital, Toronto, Ontario, Canada; 5 Department of Surgery, University of Toronto, Toronto, Ontario, Canada; 6 Ontario Institute for Cancer Research, Cancer Stem Cell Program, Toronto, Ontario, Canada; Baylor College of Medicine, United States of America

## Abstract

Sarcomas are a heterogeneous group of mesenchymal malignancies and unfortunately there are limited functional genomics platforms to assess the molecular pathways contributing to sarcomagenesis. Thus, novel model systems are needed to validate which genes should be targeted for therapeutic intervention. We hypothesized that delivery of oncogenes into mouse skeletal muscle using a retroviral (RCAS-TVA) system would result in sarcomagenesis. We also sought to determine if the cell type transformed (mesenchymal progenitors vs. terminally differentiated tissues) would influence sarcoma biology. Cells transduced with RCAS vectors directing the expression of oncoproteins Kras^G12D^, c-Myc and/or Igf2 were injected into the hindlimbs of mice that expressed the retroviral TVA receptor in neural/mesenchymal progenitors, skeletal/cardiac muscle or ubiquitously (N-tva, AKE and BKE strains respectively). Disrupting the G1 checkpoint CDKN2 (*p16/p19^−/−^*) resulted in sarcoma in 30% of *p16/p19^−/−^*xN-tva mice with a median latency of 23 weeks (range 8–40 weeks). A similar incidence occurred in *p16/p19^−/−^*xBKE mice (32%), however, a shorter median latency (10.4 weeks) was observed. *p16/p19^−/−^*xAKE mice also developed sarcomas (24% incidence; median 9 weeks) yet 31% of mice also developed lung sarcomas. Gene-anchored PCR demonstrated retroviral DNA integration in 86% of N-tva, 93% of BKE and 88% of AKE tumors. Kras^G12D^ was the most frequent oncogene isolated. Oncogene delivery by the RCAS-TVA system can generate sarcomas in mice with a defective cell cycle checkpoint. Sarcoma biology differed between the different RCAS models we created, likely due to the cell population being transformed. This genetically flexible system will be a valuable tool for sarcoma research.

## Introduction

Soft tissue sarcomas (STS) represent a heterogeneous group of mesenchymal tumors whose molecular genetics can be broadly divided into two categories [1]. The first group is characterized by simple karyotypic defects that include recurrent chromosomal translocations and oncogenic mutations. These findings can be exploited for diagnostic confirmation and are often central to tumorigenesis. The second group is genomically unstable, resulting in complex cytogenetic lesions that characteristically harbor multiple genetic alterations, including cell cycle checkpoint mutations such as p53, CDKN2A and RB1[1]. With the recent publication of large-scale genomic alterations in sarcoma, it is important to be able to efficiently identify genes contributing to sarcomagenesis [2]–[4]. However, due to a lack of functional genomics platforms for cancer gene validation there is a knowledge gap in determining which genetic events should be targeted for successful therapeutic intervention.

A powerful method to determine the oncogenic potential of candidate cancer genes is through the application of animal models [5]. Current models of sarcoma include zebrafish - an elegant genetic system that has many advantages such as rapid generation of mutants and ease of live animal imaging, but may not faithfully recapitulate sarcomagenesis in mammals [6]. Genetically-engineered mice are a widely accepted mammalian model; however, this system can be limited by the number of mutations that can be introduced in a single strain. Since most human cancers arise from at least 8–10 genetic aberrations, the generation of mouse models that have the capacity to assess pools of candidate genes would be advantageous [7], [8].

**Figure 1:**
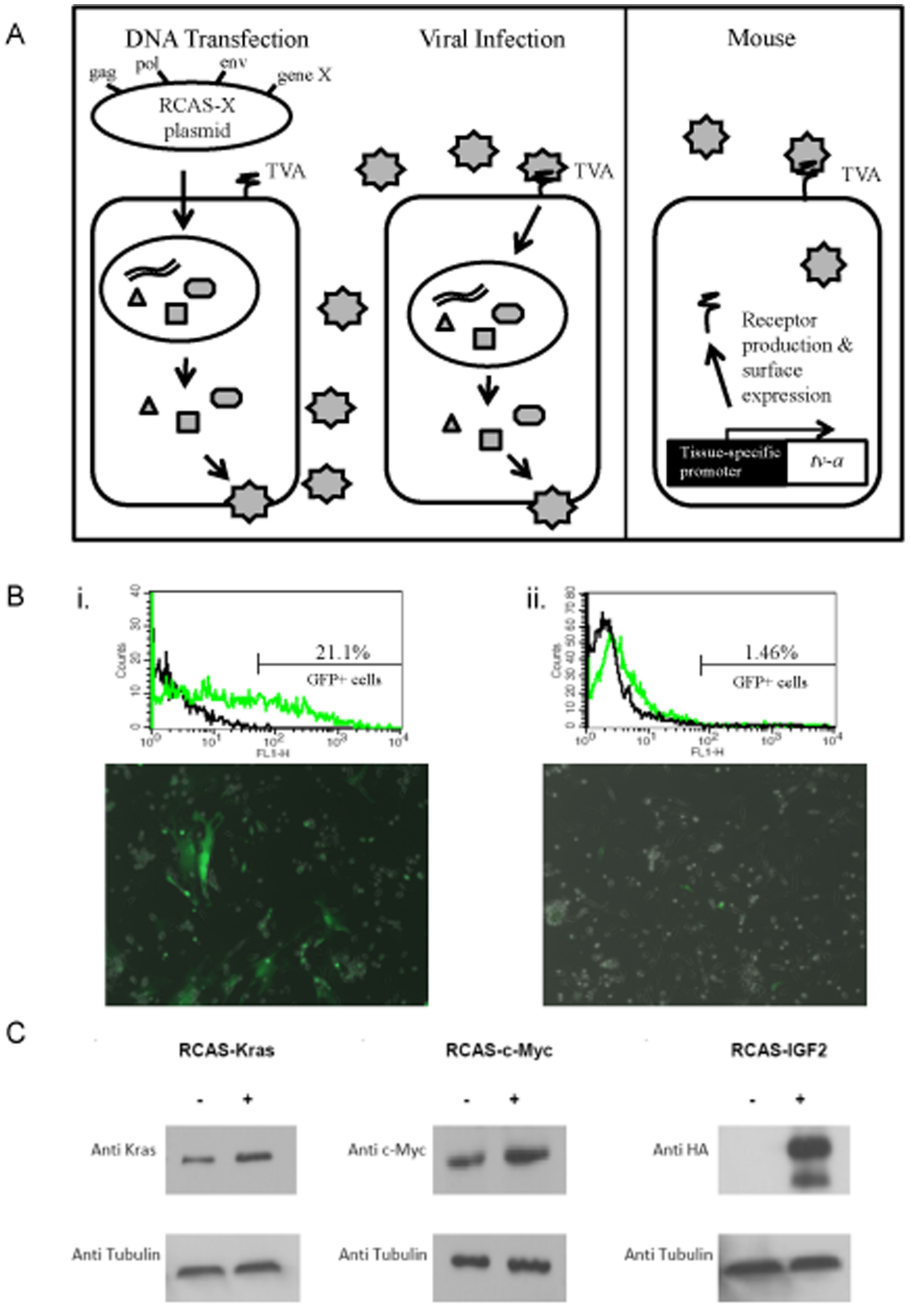
RCAS-TVA System is effective in gene delivery to neonatal hindlimbs. (**A**) In the RCAS-TVA system, DF1 chicken fibroblast cells are transfected with RCAS vector containing gene of interest (gene X). The virus produced is released into the culture medium where it infects remaining cells through endogenous TVA receptor uptake. Transgenic mice expressing the TVA receptor under a tissue-specific promoter allows for selection of which cell types are susceptible to RCAS infection and transformation. Infection of mouse cells with RCAS vector results in viral DNA integration and production of gene X's protein product. Mammalian cells do not translate the remaining viral proteins, which leaves the TVA receptor unoccupied allowing further infection of the mammalian cell by other RCAS vectors. (**B**) (i) Cells isolated from the hindlimbs of *p16/p19^−/−^*xAKE mice are infected with RCAS-GFP in culture (100× magnification) and GFP expression was analysed by FACS. (ii) GFP positive cells are identified following injection of neonatal mice and subsequent harvest 7 days later. Cells were isolated and allowed to adhere to a 10 cm plate for 3 days at which time cells were imaged (100× magnification) and analysed for GFP expression. (**C**) Protein lysates from DF1 cells transfected (+) with (i) RCAS-Kras^G12D^, (ii) RCAS-c-Myc, or (iii) RCAS-Igf2 or untransfected (-) were probed with antibodies to confirm protein expression of oncogenes of interest prior to retroviral gene delivery in neonatal hindlimbs.

The RCAS-TVA system uses the subgroup A avian leukosis virus receptor, *tva*, and a replication competent ASLV long terminal repeat with a splice acceptor (RCAS)[9]. This modified retrovirus allows for a gene of interest to be substituted for the native *src* gene. Since mammalian cells lack endogenous *tva*, this is a highly selective system as only cells that express the *tva* receptor can be infected by the RCAS vectors carrying genes of interest (Figure 1A). Thus, by creating mouse strains that are transgenic for *tva* under the control of tissue-specific promoters, it is possible to regulate which cells are infected and transformed. RCAS-TVA in vivo gene transfer has been successfully used to generate several types of solid tumors, and the advantages and limitations of this system are well documented [9], [10]. Notably, the RCAS-TVA system can be used to introduce multiple genetic alterations simultaneously or sequentially using repeated infections [10].

In this study, we used the RCAS-TVA retrovirus system to introduce known sarcoma oncogenes Kras^G12D^, c-Myc and/or Igf2 into neonatal mouse hindlimbs to determine if this genetically flexible system would result in tumorigenesis. Oncogene delivery resulted in the generation of soft tissue sarcomas; however this was dependent upon abrogation of the p16/p19 tumor suppressor locus. Interestingly, the use of different promoters for the *tva* receptor appeared to have some influence over the differentiation of the tumors and the development of sarcoma distant to the injection site.

## Materials and Methods

### Tva Mouse Strains and Genotyping

Three strains of mice were used to develop RCAS-TVA sarcoma models; AKE, BKE, and N-tva [11]-[13]. AKE and BKE mice were kindly provided by Dr. Steven Hughes, National Cancer Institute, while the *p16/p19^−/−^* and N-tva mice were a generous gift of Dr. Eric Holland, Sloan-Kettering Research Institute. Mice were housed in conventional conditions and experiments were carried out with the approval of the Animal Care Committee at the Toronto Centre for Phenogenomics (Protocol Number: 0135a-H). Animals were genotyped using PCR to detect the presence of the *tva* transgene *p16/p19* alleles as previously described [11], [14].

### RCAS Vectors and Production of Virus-Producing DF1 Cells

RCAS retroviral vectors containing cDNA for *Kras^G12D^* (RCAS-Kras^G12D^), *c-Myc* (RCAS-c-Myc), green fluorescent protein (RCAS-GFP), as well as empty vector RCAS virus (RCAS-Y) were provided by Dr. E. Holland, while the RCAS vector containing haemagglutinin (HA)-tagged *Igf2* (RCAS-Igf2) was kindly provided by Dr. Dan Fults (University of Utah). DF1 chicken fibroblasts cells were transfected, cultured and processed as previously described [15]. Myoblasts were harvested and cultured using standard conditions [16]. Immunoblot analysis of DF1 cells was performed to assess expression of RCAS-Kras^G12D^ and RCAS-c-Myc; using pan-Ras (1∶100, Calbiochem) and c-Myc 9E10 (1∶2000, generously provided by Dr. Linda Penn at the Ontario Cancer Institute), respectively. Igf2 expression was detected by probing for HA Tag using Y-11 (1∶1000, Santa Cruz Biotechnology). Tubulin was detected with α-tubulin DM1A (1∶1000, Sigma-Aldrich Corp). Secondary antibody (anti-mouse IgG from GE Healthcare or anti-rabbit IgG from Jackson Immunoresearch) was applied at a dilution of 1∶5000.

### *In Vitro* and *In Vivo* Evaluation Gene Delivery in the RCAS-TVA System

An in vitro assay was performed in cells derived from skeletal muscle harvested from 1–2 day old *p16/p19^−/−^*xAKE, *p16/p19^−/−^*xBKE and *p16/p19^−/−^*xN-tva mice as previously described [18]. Cells were serially passaged (n = 3) using a fibroblast depletion protocol. Viral supernatant from DF1 cells transfected with GFP or Empty Vector (EV) was centrifuged and filtered. 2 ml of viral supernatant was added to 4 ml fresh myoblast harvest media. Cells were transfected for 24 hrs and then the media was changed. Transfected cells were imaged for GFP positivity 72 hours later with an Olympus IX51 microscope using QCapturePro 6.0 software and then were harvested for FACS and/or PCR analysis.

1–2 day old *p16/p19^−/−^*xAKE, *p16/p19^−/−^*xBKE or *p16/p19^−/−^*xN-tva mouse pups were injected with GFP or EV expressing DF-1 cells in both hind limbs. The injection site was marked with the permanent tattoo, Endospot. Mice were aged for 7 days at which time skeletal muscle was harvested based on identifying the injection site. Cells were plated in p100 collagen-coated dishes and were imaged 72 hours later for GFP expression as previously described and harvested for FACS and/or PCR analysis.

### Introduction of RCAS vectors into Transgenic Mice

DF1 cells infected with RCAS-Kras^G12D^, RCAS-c-Myc, RCAS-Igf2 or RCAS-GFP were resuspended in DMEM at a concentration of 250 cells/µl. A 4 uL mixture of DF1 chicken fibroblast cells containing oncogenic RCAS vectors was injected into the left hindlimb of neonatal mice. An empty RCAS vector was similarly injected as a control for random retrovirus insertion. Experimental animals were monitored daily for overall health, as well as weekly tumor evaluation.

### Tumor Harvest and Pathological Assessment

Mice were sacrificed according to animal facility standard operating procedures for tumors measuring 1.5 cm or other limitations to quality of life including respiratory compromise or malnourishment. Necropsy was performed and tumor, lung tissue and normal tissues were harvested and fixed in 10% neutral buffered formalin for histological analysis, and snap frozen in liquid nitrogen for molecular studies. Fixed tissues were paraffin-embedded and cut into 4 µm sections. Hematoxylin and eosin was performed by routine techniques. Conventional immunohistochemistry was performed using: mouse anti-desmin (clone D33, 1∶100, Dako Canada Inc), mouse anti-SMA (clone 1A4, 1∶100, Dako), rabbit anti-S100B (1∶100, Dako), MyoD1 (clone 5.8A, 1∶100, Dako), mouse-cytokeratin clone AE3 (1∶50, EMD Millipore), rabbit anti-IGF2 (1∶100 abcam, Toronto, ON), rat anti-CD34 (1∶50, abcam), rat anti-CD45 (clone 30-F11, 1∶50, Becton Dickinson) and rabbit anti-Ki67 (clone SP6, 1∶100, Thermo Scientific). Sections were incubated overnight at 4°C followed by incubation with a biotinylated secondary antibody, 1∶250, for 1 hour. M.O.M Peroxidase kit (Vector Laboratories) was used for mouse-on-mouse reactions, all others antibodies were detected with the Vectastain Elite ABC kit. Visualization was performed using 3,3′-diaminobendzidine and counterstained with hematoxylin. All sections were reviewed in collaboration with a dedicated sarcoma pathologist at Mount Sinai Hospital. Staining density was scored using proportion and intensity measures as described [19].

### RCAS Vector PCR

To determine if RCAS vectors were present in the tumors generated, gene-anchored PCR was performed. DNA was extracted from snap-frozen tumor samples using DNeasy Blood and Tissue Kit (Qiagen) or from paraffin-embedded slides using the Pinpoint Slide DNA Isolation System (Zymo Research). PCR was performed using a 5′ primer specific to the RCAS viral vector and a 3′ primer specific to one of the oncogene/growth factors injected as follows: RCASF, 5′-TGAGCTGACTCTGCTGGTGG-3′; KrasR, 5′-GGGTCGTACTCATCCACAAAG-3′; c-MycR, 5′-CTCGTCGCAGTAGAAATACGG-3′; Igf2R, 5′-TGAAGCGTGTCAACAAGCTCC-3′. Each 25 uL reaction mix contained 100 ng DNA, 19.6 uL water, 2.5 uL 10X Thermopol buffer (New England BioLabs, Ipswich, MA), 0.2 mM deoxyribonucleotide triphosphate (dNTP), 0.5 uM each of forward and reverse primer, 2 U Taq DNA Polymerase (NEB). The PCR protocol utilized was: 95°C ×5 min, (95°C ×30 sec, 62°C ×30 sec, 72°C ×60 sec) ×30 cycles, 72°C ×5 min. Products were separated on a 1% agarose gel, stained with ethidium bromide and imaged under ultraviolet light. The RCAS-Kras^G12D^, RCAS-c-Myc, RCAS-Igf2 vectors were identified by 600 bp, 500 bp, and 550 bp bands, respectively.

### Species-specific PCR

We performed a PCR-based strategy for estimating the contributions of each species, mouse vs. chicken DF-1 cells in our model [17]. RNA was isolated using the Purelink RNA mini kit (Life Technologies). Concentration and purity of RNA was assessed using the Nanodrop-8000 Spectrophotometer (Thermo Scientific). 250 ng of total RNA was converted into cDNA using a 15 µl reaction with qScript (VWR). The C1000 thermal cycler (Bio-Rad Laboratories) was programmed as follows: 22°C for 5 m, 42°C for 30 m, 85°C for 5 m, and a 4°C hold. Primers against RPL19 were obtained from Eurofins MWG Operon (Huntsville, AL). Species-specific primers have an amplicon length of 83 bp, while the universal primers are 127 bp (see Figure S2B for primers). 2 ul of cDNA was added to Taq DNA Polymerase with ThermoPol Buffer (NEB,), DMSO, MgCl_2_, dNTPs and RNase-free dH_2_0 for a 25 µl reaction. The thermal cycler was programmed as: 95°C for 3 m, (95°C for 30 s, 59°C for 30 s, 72°C for 60 s) ×34, 72°C for 5 m, and 4°C hold. Samples were visualized on a 2% agarose gel using SYBR Safe DNA gel stain (Life Technologies).

### Statistical Analysis

Survival curves were calculated using the Kaplan-Meier method and were generated using SPSS (IBM, version 19).

## Results

### The RCAS-TVA system is effective in gene delivery to neonatal muscle

The RCAS-TVA system has been previously utilized to create pre-clinical models of solid tumors (Figure 1A) [20], [21]. We studied transgenic mice that express tva under the control of the promoters for nestin (neural and mesenchymal progenitors), α-actin (skeletal/cardiac striated muscle) and β-actin (essentially all cells) to determine if certain cell types/developmental stages would be more amenable to tumorigenesis [11]–[13]. Since the CDKN2 locus, which encodes p16 (INK4a) and p19 (ARF) genes, has been implicated in human and murine sarcoma pathogenesis, we also sought to determine if dysregulation of this tumor suppressor would play a cooperative role in sarcomagenesis in the RCAS-TVA system [14], [22]. We first evaluated whether cells isolated from *p16/p19^−/−^*xAKE mice could be infected by RCAS vectors. Primary cell lines were created from 1 or 2 day old skeletal muscle harvested from hindlimbs and grown in supernatant containing RCAS-GFP virus. After in vitro infection with RCAS-GFP following 3 passages, approximately 21% of the plated cells expressed GFP (Figure 1Bi). Furthermore, following in vivo injection of DF-1 cells transduced with RCAS-GFP vector into *p16/p19^−/−^*xAKE mouse hindlimbs, cells were harvested 7 days later. After plating the cells (n = 3), ∼1.5% of skeletal muscle cells harvested were GFP^+^ (Figure 1Bii), demonstrating that GFP+ cells could be identified early after injection. Similarly, in *p16/p19^+/−^*xN-tva mice and *p16/p19^−/−^*xBKE mice an average of 7.3% and 3.3% of cells respectively were GFP^+^ (data not shown). Collectively, these experiments demonstrate that retroviral infection of neonatal skeletal muscle cells in *p16/p19^−/−^*xN-tva, *p16/p19^−/−^*xAKE and *p16/p19* ^−*/*−^xBKE mice is successful using RCAS vectors.

### RCAS-TVA system can be used to generate murine models of STS but requires tumor suppressor deficiency along with oncogene delivery

AKE (α-actin promoter) and BKE (β-actin promoter) mice were injected with a combination of DF-1 cells expressing c-Myc, Kras^G12D^ and Igf2 (Figure 1C). A total of 88 AKE mice were injected but did not develop tumors after a period of observation (median 89 weeks, range 82–95 weeks) (Table 1). Similarly, none of the 61 BKE mice injected with transduced DF-1 cells developed neoplasms (median 67 weeks, range 51–81 weeks). Furthermore, we injected 20 AKE and BKE mice with RCAS-c-Myc transduced cells alone (data not shown) but after a median observation of 75 weeks (range 60–90 weeks) no tumors were identified. Thus, the delivery of oncogenes and/or growth factors alone into skeletal muscle appears insufficient for sarcomagenesis in this model system.

**Table 1:** Sarcoma development occurs at injection site in *p16/p19*-deficient *tva* mice following retrovirus oncogene delivery.

Mouse strain; RCAS vectors delivered	# of mice injected	# of sarcomas at injection site (median latency)	# of sarcomas at a distant site (median latency)
*p16/p19^+/+^*xAKE; Kras^G12D^, c-Myc, Igf2	88	0	0
*p16/p19^−/−^*xAKE; Kras^G12D^, Igf2	67	16 (9 weeks)	5 (7.1 weeks)
*p16/p19^+/+^*xBKE; Kras^G12D^, c-Myc, Igf2	61	0	0
*p16/p19^−/−^*xBKE; Kras^G12D^, Igf2	44	14 (10.4 weeks)	1 (6.9 weeks)
*p16/p19^−/−^*xN-tva; Kras^G12D^, c-Myc, Igf2	74	22 (23 weeks)	0
*p16/p19^−/−^*xN-tva; empty vector	20	0	0

Pulmonary sarcoma occurred in 12% of the affected mice.

Dysregulation of the CDKN2A locus, due to either mutation or methylation, which encodes p16 and p19, has been reported in several subtypes of human sarcomas; consequently we examined if abrogation of this tumor suppressor in the RCAS-TVA system would promote sarcoma formation [23], [24]. We also sought to determine if oncogene delivery to mesenchymal and neural progenitors (N-tva), or terminally differentiated tissues (AKE and BKE) would impact sarcomagenesis. Thus, we delivered a mixture of DF-1 producing RCAS-Kras^G12D^, RCAS-c-Myc, and/or RCAS-Igf2 cells into the left hindlimbs of *p16/p19^−/−^*xN-tva, *p16/p19^−/−^*xAKE or *p16/p19^−/−^*xBKE strains.

**Figure 2:**
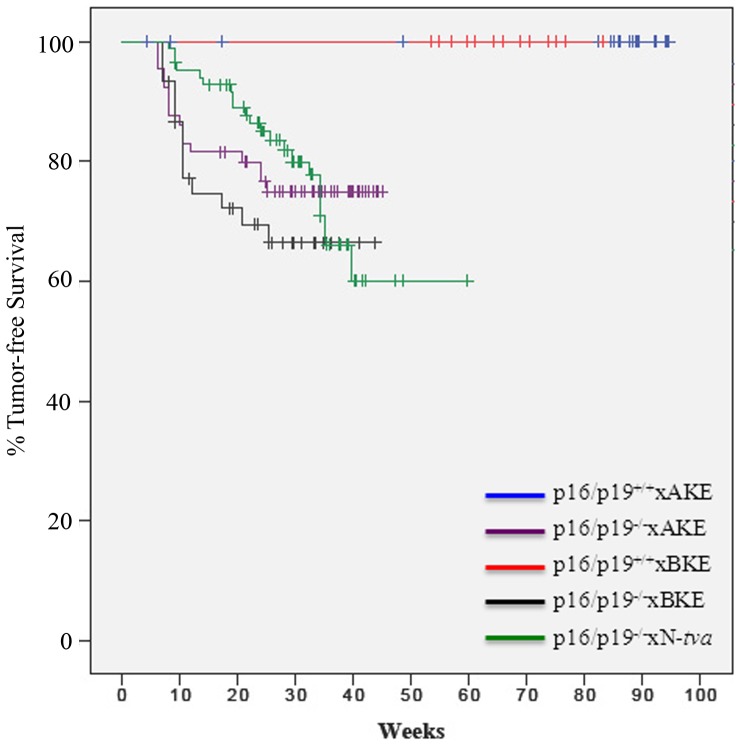
Introduction of RCAS vectors induces sarcoma in *p16/p19*-deficient *tva* mouse strains. A Kaplan-Meier analysis of mortality due to tumor formation at the RCAS injection site of *p16/p19^−/−^*x AKE, *p16/p19^−/−^*x BKE and *p16/p19^−/−^*x N-*tva* mice. Mice were euthanized and tissues were removed for histopathologic assessment to confirm sarcoma formation, assess subtype and to confirm the presence of RCAS vector. Wild-type AKE and BKE mice did not develop RCAS-associated tumors.

Of the 74 *p16/p19^−/−^*xN-tva mice injected, 22 (30%) developed soft tissue sarcoma at, or around, the injection site with a median onset of 23 weeks (range 8–36 weeks) (Table 1 and Figure 2). The tumors were white-tan, firm and circumscribed without lymph node metastasis. An additional 10 (13.5%) mice developed a tumor immediately cephalid to the left hindlimb with a median latency of 24 weeks (range 16–32 weeks). There was no gross evidence of metastatic disease in the lungs or liver. Control mice injected with RCAS-GFP or with empty RCAS vector (RCAS-Y) in their hindlimbs (n = 20) and observed for at least 38 weeks did not develop tumors (Table 1). An additional 8 (10.8%) *p16/p19^−/−^*xN-tva mice injected with oncogenic RCAS vectors developed tumors at sites distant from the experimental site with a median latency of 23 weeks (range 15–30 weeks). These tumors were considered to be spontaneous based on tumor location and histopathologic attributes, and were therefore attributed to the *p16/p19* null background of the experimental mice as previously reported [25].

We next evaluated if STS would arise in *p16/p19^−/−^*×BKE mice that constitutively expressed the *tva* receptor in almost all tissues [13]. Interestingly, we observed a shorter latency of tumor formation - 10.4 weeks (range: 7–25 weeks) but a similar incidence as 14 of 44 *p16/p19^−/−^*×BKE mice (32%) developed tumors in the left hindimb when injected with DF-1 cells expressing Kras^G12D^ and Igf2. One animal developed distant pulmonary sarcoma (Table 1). Twenty-one mice developed tumors in regions distinct from the left hindlimb (head and neck, thymus, spleen), which are likely due to spontaneous *p16/p19^−/−^* tumor formation (latency of 30 weeks; range 23–44 weeks).

Although *p16/p19^−/−^*×AKE mice had a similar incidence (24%; 16/67 animals) and latency of tumor formation (median 9 weeks, range 6–25 weeks) to the *p16/p19^−/−^*×BKE strain, a striking increase in sarcoma formation distant to the injection site was observed. Pulmonary sarcoma was observed in 5/16 (31%) of *p16/p19^−/−^*×AKE mice injected with RCAS-Kras^G12D^ and RCAS-Igf2 vectors (Table 1) in their hindlimb. In this subgroup, 3 animals developed lung sarcoma with a primary limb tumor (median latency 14.7 weeks) and two other mice had lung sarcoma only (median latency of 7.5 weeks) without detection of a primary extremity sarcoma.

### Effect of different tva promoters on tumor differentiation

**Figure 3:**
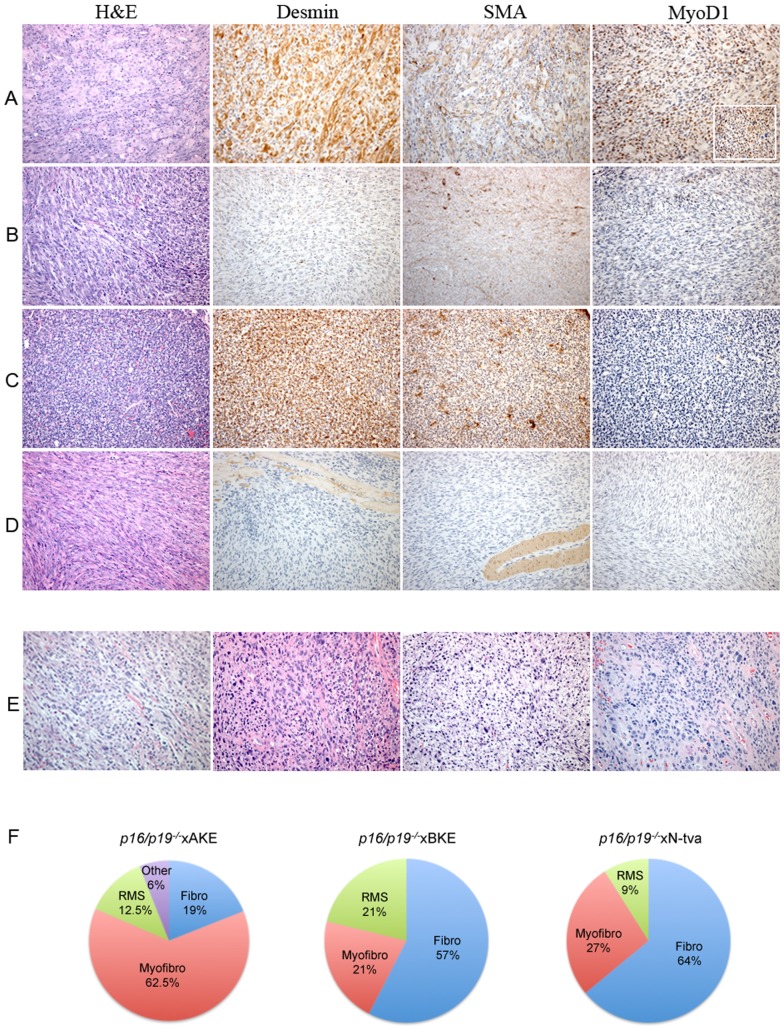
The RCAS-TVA System Produces Multiple STS Immunophenotypes. (**A**) Immunohistochemistry in a *p16/p19^−/−^*xAKE leg mass has positive tumor cell staining for desmin, SMA and MyoD1 consistent with rhabdomyosarcomatous differentiation. (**B**) A leg sarcoma in a *p16/p19^−/−^*xAKE mouse is SMA positive, consistent with myofibroblastic differentiation. (**C**) The corresponding lung sarcoma from (**B**). (**D**) The sarcoma seen in a *p16/p19^−/−^*xBKE limb has negative IHC and fascicular arrangements of cells consistent with fibrosarcomatous differentiation. (**E**) H&E stains of four human sarcomas demonstrates similar morphology to RCAS-TVA induced murine tumors. All images at ×200 magnification, except (**A**) MyoD1 insert x400. (**F**) In *p16/p19^−/−^*xAKE mice, myofibrosarcoma was the predominant STS seen in tumors that arose under the α-actin promoter. A high proportion of fibrosarcoma was seen in *p16/p19^−/−^*xBKE and *p16/p19^−/−^*xN-*tva* mice. Myofibro = Myofibrosaroma, RMS = rhabdomyosarcoma and Fibro = fibrosarcoma. Other included round cell tumour.

Histopathologic assessment was performed to classify tumours, and assess whether this was influenced by the *tva* promoters utilized. Grossly, the tumors were circumscribed and composed of sheets of cohesive spindle-to-epithelioid cells with a fascicular-to-herringbone pattern (Figure 3A–D). Rarely were areas with rhabdoid morphology noted. The cytoplasm was abundant and eosinophilic. The nuclei were ovoid and hyperchromatic, with moderate pleomorphism; mitotic activity was generally brisk (>20 per 10 high power fields) and also included ‘atypical’ figures. Tumors had a Ki67^+^ index of >30% (Figure S1). Necrosis was frequently observed. A mild, predominantly lymphoplasmacytic, inflammatory infiltrate occasionally accompanied tumours. Notably, none of the tumours exhibited epithelial differentiation or pigment production; furthermore, none of the tumours significantly expressed keratin, S100, or CD45, thereby respectively making the possibility of epithelial, neural crest and/or haematolymphoid derivation unlikely. This was confirmed by ultrastructural examination, where electron microscopy revealed the presence of intracytoplasmic filaments and rare junctions, but no features to support epithelial, neural crest or hematolymphoid differentiation (data not shown). The combined morphologic, ultrastructural and immunohistochemical findings supported a malignant mesenchymal, namely sarcomatous, origin consistent with human sarcomas (Figure 3E).

The sarcomas were pleomorphic and morphologically undifferentiated. A conventional mesenchymal immunohistochemical panel – including desmin, smooth muscle actin (SMA), MyoD1 and CD34 expression (Figure 3A–D), was used in an effort to subclassify tumors. Overall the most common immunophenotype was a lack of expression of all tested markers (48%); this immunohistochemical phenotype, combined with the spindle cell morphology and ultrastructural findings, is similar to fibrosarcoma in humans, and therefore suggestive of fibrosarcomatous differentiation. Other immunohistochemical phenotypes observed in our models included myofibroblastic differentiation (SMA^+^, desmin^+/*−*^), rhabdomyosarcomatous differentiation (MyoD1^+^, desmin^+/*−*^, SMA^+/*−*^) and an undifferentiated round cell tumor (Figure 3F). We hypothesized that rhabdomyosarcomatous differentiation (RMS) might predominate in AKE mice due to *tva* expression in terminally differentiated skeletal muscle. However, sarcomas with myofibroblastic differentiation were most commonly observed (62.5%) with only 12.5% of AKE tumors bearing evidence of RMS-type differentiation (Figure 3A). Disease distant to the injection site, which could represent metastasis, appeared restricted to the lungs. Pulmonary sarcomas were grossly circumscribed and often multifocal. Histologically, they tended to show morphologic and immunohistochemical overlap with the primary tumour (Figure 3F).

### Integration of RCAS DNA in the majority of tva murine sarcomas

Gene-anchored PCR to determine if RCAS vector DNA (RCAS-Kras^G12D^, RCAS-c-Myc and/or RCAS-Igf2) was integrated into the host genome was performed in all tumors (Table 2). At least 1 vector amplicon was detected in 19 of 22 (86%) N-tva, 13 of 14 (93%) BKE and 14 of 16 (88%) AKE sarcomas. Half of the pulmonary metastases had at least one RCAS vector detected (1 BKE and 2 AKE mice with pulmonary disease only). RCAS-Kras^G12D^ was the most common vector detected in 40/52 (77%) of all RCAS induced-sarcomas. Multiple RCAS vectors were detected in 50% of samples. Most frequently Kras and Igf2 were identified concomitantly (9/12 AKE, 10/13 BKE). In a small proportion of N-tva sarcomas, all three vectors were detected (9%). Sarcoma differentiation did not appear to correlate with the RCAS vectors identified. The majority of flank tumors (10 N-tva and 3 AKE mice) contained one or more RCAS vectors (69%), suggesting that they were indeed the result of RCAS-mediated gene delivery. In contrast, RCAS amplicons were not detected in normal skeletal muscle or spontaneous tumors. Finally, species-specific PCR was performed, which confirmed there was no chicken genome present in RCAS-induced tumors (Figure S2A). Thus, the sarcomas generated from this model system likely arose from viral transmission of oncogenes and not from oncogenic transformation of DF-1 cells themselves.

**Table 2:** Integration of RCAS DNA in the majority of RCAS-TVA induced sarcomas.

RCAS Vector Detected	*p16/p19^−/−^*xN-tva (n = 22)	*p16/p19^−/−^*xBKE (n = 14)		*p16/p19^−/−^*xAKE (n = 16)	
		Limb (n = 14)	Lung (n = 1)	Limb (n = 14)	Lung (n = 5)
Multiple vectors	7 (32%)	10 (71%)	1 (100%)	9 (64%)	0 (0%)
RCAS-Kras^G12D^	13 (59%)	13 (93%)	1 (100%)	12 (86%)	2 (40%)
RCAS-Igf2	6 (27%)	10 (71%)	1 (100%)	9 (64%)	0 (0%)
RCAS-cMyc	9 (41%)	-	-	-	-
No vector	3 (14%)	1 (7%)	0	2 (14%)	3 (60%)

Gene anchored PCR was performed to detect presence of RCAS DNA within harvested tumor specimens.

No RCAS DNA was detected in spontaneous tumors that arose in mice with *p16/p19-*deficiency only.

## Discussion

AKE and BKE mice with functional CDKN2 (*p16/p19^+/+^*) did not develop sarcomas following injection with RCAS vectors carrying known oncogenes (Table 1; Figure 2). Although AKE and BKE mice may form tumors beyond our observation period of at least 1.5 years, it is also possible that the introduction of oncogenes alone into the hindlimbs of mice is insufficient for sarcoma development using the RCAS-TVA system. This is supported by the fact that sarcomas were readily generated at RCAS injection sites in the three *p16/p19*-deficient *tva* strains (Table 1; Figure 2). Thus, abrogation of a tumor suppressor is likely required for sarcomagenesis in this system. Other mouse models of sarcoma also demonstrate tumor formation is dependent on oncogene activation in conjunction with loss of a tumor suppressor pathway such as p53 or p16/p19 [14], [26]–[32]. Furthermore, the importance of these key genomic stability pathways in STS is substantiated by the high proportion of human STS that have tumor suppressor inactivation [23], [33]–[35].

No hindlimb tumors were identified in the *p16/p19*-deficient N-tva mice (n = 20) injected with empty RCAS-Y vector after a median observation of 38 weeks (range 25–45 weeks) (Table 1). Thus, the tumors in our models were likely secondary to oncogene/growth factor effects and not the result of tissue injury during injection, or a product of random viral integration into the mouse genome. Our finding that STS can be generated in mice with a deregulated tumor suppressor pathway along with the introduction of oncogenes and growth factors into hindlimb skeletal muscle is therefore consistent with previous mouse models and human sarcomas [2], [14], [31].

We identified flank sarcomas in 13 of 52 experimental animals (*p16/p19^−/−^* xN-tva; n = 10 and *p16/p19^−/−^* xAKE; n = 3). This finding could have several explanations. Firstly, flank tumors may be unrelated to RCAS vector injection all together, and could be the result of the *p16/p19*-null background. Alternatively, the injected DF1 cells or the virus they produced may have migrated from the injection area to the flank of the animal with subsequent infection at a secondary site. However, this is unlikely given that use of an RCAS-AP vector demonstrated that only *tva*-expressing cells at the injection site are infected by virus, with no alkaline phosphatase staining observed distantly on pathological analysis [9]. Finally, the cephalid location of these tumors may be the result of technical error. In our model system, the RCAS vector-producing DF1 cells are introduced into neonatal mice (day 1), which makes targeting the hindlimb challenging due to its small size. We contend that the most likely explanation for flank sarcomas is due to inadvertent RCAS vector injection as all 13 flank sarcomas were on the left side of the animal, the same side that the oncogenic vectors were introduced. No tumors were observed on the right flank. Furthermore, the majority of the flank sarcomas (69%) were found to be positive for at least one RCAS vector, suggesting that these tumors were the result of the oncogene delivery by this retroviral system. We propose that remaining 3 flank tumors, which did not contain an RCAS vector by PCR, may represent sampling error as only a small portion of each tumor was used for DNA extraction.

Metastatic disease in human STS characteristically spreads hematogenously while lymphatic involvement is uncommon in most subtypes [36]. Consistent with human disease, RCAS-TVA STS mice did not have lymph node involvement. The only possible metastatic disease documented in our mouse models was in the lungs, the most common site of metastasis in human sarcomas. In most cases, multiple pulmonary nodules were identified. Interestingly, two *p16/p19^−/−^* xAKE mice succumbed to pulmonary sarcoma within 7 weeks. While no overt mass was detected at the injection site, an occult extremity tumor may have been missed with routine sampling. Distant disease developed most frequently in the *p16/p19^−/−^* xAKE strain (31% of the affected mice) compared to *p16/p19^−/−^* xBKE (7%) and *p16/p19^−/−^* xN-tva (0). This may be due to differences in the cell type transformed, which is dictated by *tva* expression or as a result of background modifiers in the AKE strain itself. Further work to more globally compare injection site sarcomas and distant disease using expression analysis may help identify co-operative mutations that promote tumor dissemination in sarcomagenesis.

One of the strengths of the RCAS-TVA system is that it restricts the cell types that are infected by RCAS vectors. We hypothesized that *tva* expression in terminally differentiated tissue such as skeletal muscle would result in a consistent tumour type such as rhabdomyosarcoma (RMS), yet we observed morphologically undifferentiated sarcomas that had immunohistochemical evidence of myofibroblastic, rhabdomyosarcomatous or fibroblastic differentiation. Although the developmental context in which oncogenes are introduced can be critical in determining the subtype of sarcoma formed in mice, the type of oncogenic pathways that are activated will also directly impact the tumour phenotype [37], [38]. In all three mouse models we generated, Kras was the predominant oncogene isolated (77%). It is well established that constitutive RAS activation leads to dysregulated cell proliferation and blocks myogenic differentiation by downregulating key myogenic factors such as MyoD1 and myogenin [39]. Poorly differentiated sarcomas have also been reported in other Kras-overexpressing STS mouse models [27], [31], [40], [41]. Similarly, IGF2 mRNA levels have been inversely correlated with the degree of differentiation in both subtypes of RMS, however, exactly how IGF2 contributes to sarcomagenesis is still largely unknown [42]. It is also possible that oncogene expression levels are affected by where the gene of interest integrates in the host genome. Another advantage of this retroviral system is that several genes can be delivered into the same somatic cell simulatenously or sequentially in an immunocompetent microenvironment [10]. We were able to detect more than one oncogene in 50% of the sarcomas generated. In two *p16/p19^−/−^*xN-tva mice, all three oncogenes were identified. However, without single cell analysis we are unable to clarify if the oncogenes introduced infected an individual cell or if the tumors we generated represent an admixture of virally infected cells.

Although our observed tumor frequency was ∼ 30%, rapid tumour development was observed in all three strains. Future work to improve viral transfection rates in the RCAS-TVA system would likely create a more robust functional genomics platform. Tumor penetrance may also be enhanced by increasing the number or modifying the type of oncogenes delivered, and/or by abrogating other key STS tumor suppressors such as p53. With the establishment of RCAS-TVA soft tissue sarcoma mouse models, we propose that this genetically flexible system will allow us to more fully explore the genetic events that regulate both sarcoma formation and the development of distant disease, and will be an effective tool for the testing of novel therapeutic agents.

## Supporting Information

Figure S1 **Representative photomicrographs of mouse tumors demonstrate high Ki-67 proliferation indexes in all genotypes.** High Ki-67 index (>30%) indicate high rates of proliferation are present in tumor samples. Above is a subset of samples from each mouse genotype shown at 100x on left and 200× magnification on right.
(DOCX)

Figure S2 **Species-specific PCR against RPL19 indicate mouse tumors do not contain chicken DNA and thus tumor formation is driven by integration of oncogenes into the mouse genome.** (**A**). To confirm that DF1 transfected cells directly injected into mouse skeletal muscle are not oncogenic themselves and thus could drive tumor formation, we performed a PCR-based strategy for estimating the contributions of each species in xenografts [17]. RNA was isolated from 3 tumors per mouse strain, converted to cDNA and added to a PCR mix containing mouse (M), chicken (C) and/or universal (U) primers. PCR was performed as described and visualized on a 2% agarose, above is a subset of samples and the associated controls. Samples were run in triplicate. (**B**) Primers used in PCR strategy.
(DOCX)
