# Supplementary figures and images for: Development of Genetically Flexible Mouse Models of Sarcoma Using RCAS-TVA Mediated Gene Delivery
Source: PLoS One. 2014 Apr 14;9(4):e94817. doi: 10.1371/journal.pone.0094817 (PMC3986235; doi:10.1371/journal.pone.0094817)

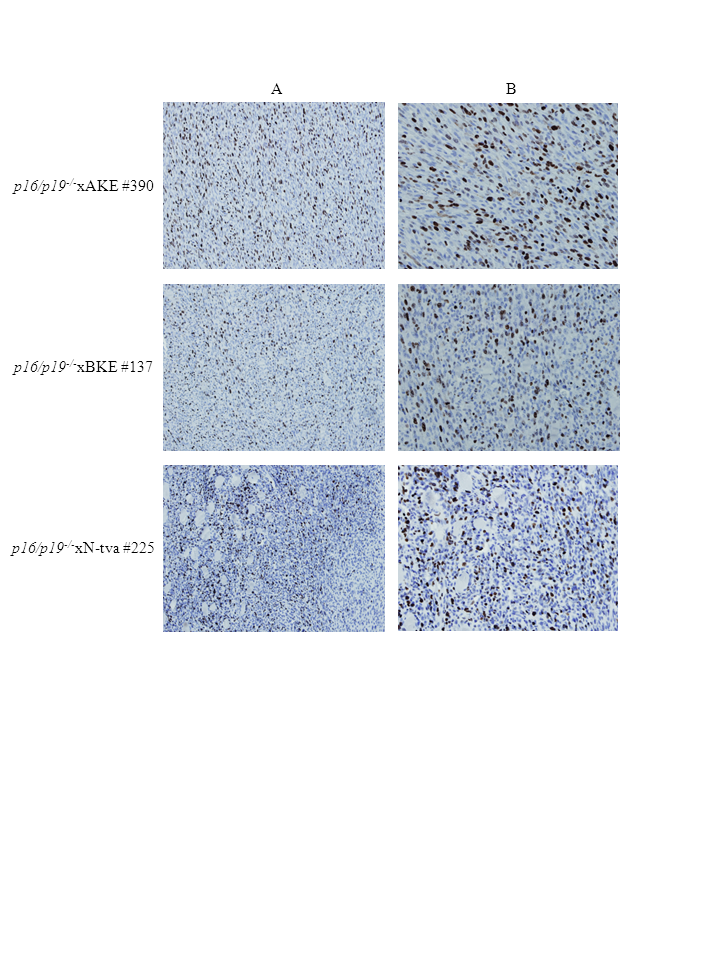

Supplement: Figure S1 — Representative photomicrographs of mouse tumors demonstrate high Ki-67 proliferation indexes in all genotypes. High Ki-67 index (>30%) indicate high rates of proliferation are present in tumor samples. Above is a subset of samples from each mouse genotype shown at 100x on left and 200× magnification on right. (DOCX) [file pone.0094817.s001.docx]
