# Supplementary material for: Development of Genetically Flexible Mouse Models of Sarcoma Using RCAS-TVA Mediated Gene Delivery
Source: PLoS One. 2014 Apr 14;9(4):e94817. doi: 10.1371/journal.pone.0094817 (PMC3986235; doi:10.1371/journal.pone.0094817)
Supplement: Figure S2 — Species-specific PCR against RPL19 indicate mouse tumors do not contain chicken DNA and thus tumor formation is driven by integration of oncogenes into the mouse genome. (A). To confirm that DF1 transfected cells directly injected into mouse skeletal muscle are not oncogenic themselves and thus could drive tumor formation, we performed a PCR-based strategy for estimating the contributions of each species in xenografts [17]. RNA was isolated from 3 tumors per mouse strain, converted to cDNA and added to a PCR mix containing mouse (M), chicken (C) and/or universal (U) primers. PCR was performed as described and visualized on a 2% agarose, above is a subset of samples and the associated controls. Samples were run in triplicate. (B) Primers used in PCR strategy. (DOCX) [file pone.0094817.s002.docx]

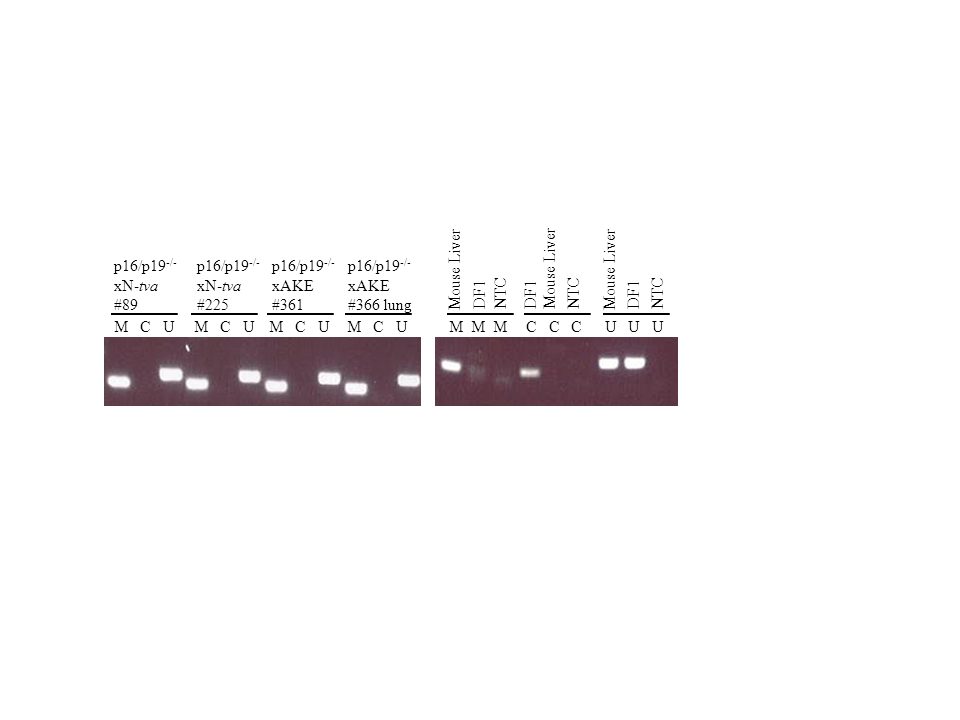


| **RPL19 primers** |  |
| --- | --- |
| Primer Name | Sequence |
| Universal - Forward | ACGCCAACTCGCGTCAGCAG |
| Universal - Reverse | ATATGCCTGCCCTTCCGGC |
| Chicken - Forward | TTGATCATCCGCAAACCCGTGAAC |
| Mouse - Forward | CTGATCATCCGCAAGCCTGTGAAT |
